# Twenty Years, and More to Come: Learning What Makes Some Transplants Ultra-Long Survivors

**DOI:** 10.3389/ti.2022.11036

**Published:** 2022-12-05

**Authors:** Umberto Maggiore

**Affiliations:** Dipartimento di Medicina e Chirurgia, UO Nefrologia, Azienda Ospedaliera-Universitaria Parma, Università di Parma, Parma, Italy

**Keywords:** kidney transplantation, graft function, long-term survival, DSA, late graft loss

Lifetime survival of the kidney graft and normal life expectancy of the recipient have always been the ultimate goals of kidney transplantation. To survive for several decades, the kidney graft must overcome multiple potential threats, including immune-mediated cell damage, ischemia/reperfusion injury-induced cell senescence and fibrosis, suboptimal nephron mass of kidneys obtained from marginal donors, primary kidney disease recurrence; chronic or recurrent graft infections, and drug nephrotoxicity. The antirejection treatment, which protects against alloimmune-mediated injury, may do so at the expense of the patient’s life expectancy. By inhibiting the immune response, anti-rejection drugs increase the risk and severity of infections and cancer. Moreover, some anti-rejection drugs can cause hypertension and severe metabolic complications such as diabetes mellitus, obesity, and hyperlipemia that augment the cardiovascular risk. Cardiovascular abnormalities developed during long exposure to dialysis (such as vascular calcification), patient frailty at time of transplantation, and the effect of long-standing previous immunosuppressive regimens used for the treatment of the primary kidney disease, may also impair long term patient survival, despite a successful transplant procedure.

Regardless of these threats, there exists a subset of kidney transplant recipients whose grafts survived for numerous years, formerly represented by young patients who recieved an azathioprine-based immunesuppressive regimen alongside kidneys from donors who died as a result of traffic accidents. Over time this population has been enriched by older patients who received standard calcineurin-based immunosuppressive regimes alongside kidneys from marginal and after-circulatory-death donors (DCD). Understanding the unique characteristics of this population may provide critical information for the management of this population.

The determinants and complications that may adversely affect ultra-long term clinical outcomes are highly heterogeneous. Some of those patients might have survived for decades without developing any relevant risk factor or complication. In contrast, others have developed relevant risk factors or complications (e.g., donor-specific antibodies), but did so too recently with respect to the start of follow-up, therefore the effect on transplant loss could not be yet detected. Some of the risk factors and complications may be still amenable to intervention even decades after transplantation. The benefit to risk ratio of therapeutic intervention at these later stages, such as immunosuppressive treatment for chronic antibody mediated rejection, should consider that decades of exposure to immunosuppression increase the susceptibility to infection and cancer, and that substantial nephron loss unavoidably takes place over decades after transplantation, irrespective of the ongoing alloimmune mediate injury, which leaves little margin for graft function recovery.

In this issue of the journal, Reimann et al. (1) report the determinants of clinical outcomes in kidney transplant recipients who entered the third decade of follow-up and who had received a transplant over a period that spanned from 1981 to 1999 which, compared to previous literature on the topic (2,3,4,5), encompasses the most recent transplantation years reported to date. This population included more recipients of suboptimal donors who were also on modern immunosuppression. After analysing 248 survivors 20 years or more post-transplant, the authors identified 96 patients (39%) who had superior graft function (defined by the joint presence of eGFR ≥45 ml/min, proteinuria ≤300 mg/day, and eGFR-slope ≤2 ml/min/1.73 m^2^/year) and who were then compared with the remaining patients. As expected, superior graft function was associated with less exposure to pre-transplant dialysis, better graft quality (younger donor age, less DCD donors), lower rates of T cell mediated rejection, and primary kidney disease not encompassing glomerulonephritis, which included diseases that can recur very late post-transplantation, such as IgA nephropathy (6). After 10 years of further follow-up, having a superior graft function was associated with a twenty-fold reduction in the rate of death censored graft failure (DC-GF). Group membership (i.e., either superior graft function, or other patients) did not apparently affect mortality rate, although the data may not have sufficient statistical power and enough length of follow-up to detect differences in mortality. Even after controlling for group membership, the presence of donor-specific antibodies was still associated with a three-fold increase in the DC-GF rate. Such findings imply that donor-specific antibodies (DSA) remain a powerful biomarker of ongoing chronic antibody-mediated rejection even several decades after transplantation. The incidence of DSA was low in this population, being approximately 20% at 20 years, which is apparently in contrast with the 20% cumulative incidence previously documented as early as 5 years post-transplantation (7). Surprisingly, the prevalence and type of DSA, and the graft immunogenicity as estimated by the PIRCHE II score (8), did not differ between the two groups. These paradoxical findings may be related to the fact that most patients were not typed for HLA-DP and -DQ and that all the assessment was based on low resolution HLA typing. An alternative, not mutually exclusive explanation, is that differences in graft function between the two groups were related to non-alloimmune mediated graft injury and that most cases of chronic rejection started only late after transplantation. Interestingly, the type of immunosuppression apparently affected clinical outcomes, steroid-free regimens being associated with approximately one-third (hazard ratio vs. steroid-based: 1/2.844 = 0.35) the DC-GF rate, and cyclosporine-based regimens being associated with approximately one-third (hazard ration vs. cyclosporine-free: 0.30) the mortality rate. However, because the hazard ratio estimates were not fully adjusted for potential confounding factors, they can hardly be interpreted as expressing a causal relationship between immunosuppressive regimen and clinical outcomes. Rather, they may be regarded as markers of a better prognostic profile. It is interesting to note that even beyond 2 decades post-transplantation, older donor age remains associated with increased DC-GF rate. In fact, the hazard ratio of DC-GF was 1.032 per 1 year age increase. This may seem trivial, but it corresponds to almost twice the DC-GF rate when comparing donor age differences of 20 years (1.032 to the power of 20 = 1.88) and it is especially relevant as the donor age distribution varied widely, ranging between 3 and 72 years (median 32). Although older donor age is associated with inferior outcomes, the survival advantage of transplantation over remaining on the wating list still holds true for kidneys from elderly donors (9,10). Likely, this benefit (although not formally tested) was particularly high in ultra-long survivor transplant recipients.

Given the growth of the ultra-long survivor population and its heterogeneous profile, the study from Reimann et al. (1) provides novel information that may help to guide decision making in their clinical management.
